# Immunocytochemical characterization of *ex vivo* cultured conjunctival explants; marker validation for the identification of squamous epithelial cells and goblet cells

**DOI:** 10.3389/fmed.2023.1024926

**Published:** 2023-02-27

**Authors:** Sara I. Van Acker, Bert Van den Bogerd, Michel Haagdorens, Carina Koppen, Isabel Pintelon

**Affiliations:** ^1^Antwerp Research Group for Ocular Science, Translational Neurosciences, Faculty of Medicine and Health Sciences, University of Antwerp, Wilrijk, Belgium; ^2^Department of Ophthalmology, Antwerp University Hospital, Edegem, Belgium; ^3^Laboratory of Cell Biology and Histology, Faculty of Pharmaceutical, Biomedical and Veterinary Sciences, University of Antwerp, Wilrijk, Belgium

**Keywords:** conjunctiva, immunocytochemistry, mucin, keratin, squamous epithelial cells, goblet cells

## Abstract

Tissue-engineered products are at the cutting edge of innovation considering their potential to functionally and structurally repair various tissue defects when the body’s own regenerative capacity is exhausted. At the ocular surface, the wound healing response to extensive conjunctival damage results in tissue repair with structural alterations or permanent scar formation rather than regeneration of the physiological conjunctiva. Conjunctival tissue engineering therefore represents a promising therapeutic option to reconstruct the ocular surface in severe cicatrizing pathologies. During the rapid race to be a pioneer, it seems that one of the fundamental steps of tissue engineering has been neglected; a proper cellular characterization of the tissue-engineered equivalents, both morphologically and functionally. Currently, no consensus has been reached on an identification strategy and/or markers for the characterization of cultured squamous epithelial and goblet cells. This study therefore evaluated the accuracy of promising markers to identify differentiated conjunctival-derived cells in human primary explant cultures through immunocytochemistry, including keratins (i.e., K7, K13, and K19) and mucins (i.e., MUC1, MUC5AC, and PAS-positivity). Comparison of the *in vivo* and *in vitro* cellular profiles revealed that the widely used goblet cell marker K7 does not function adequately in an *in vitro* setting. The other investigated markers offer a powerful tool to distinguish cultured squamous epithelial cells (i.e., MUC1 and K13), goblet cells (i.e., MUC5AC and PAS-staining), and conjunctival-derived cells in general (i.e., K19). In conclusion, this study emphasizes the power alongside potential pitfalls of conjunctival markers to assess the clinical safety and efficacy of conjunctival tissue-engineered products.

## 1. Introduction

Tissue-engineered products are developed with the ultimate goal of replacing damaged tissue with a functional equivalent structure. The inclusion of living cells renders tissue-engineered products among the most complex pharmaceuticals as cellular behavior depends on the microenvironment and interactions (1). When cells are removed from their *in vivo* environment and placed into culture, their phenotype, migration pattern, and other characteristics can change accordingly (1). An exceptionally powerful tool to assess if cultured cells retain their *in vivo* phenotype is through the immunocytochemical detection and localization of cell-specific proteins – preferably linked to their *in vivo* functionality – at subcellular, cellular, and even tissue levels (2, 3).

The phenotypic identification of various cell types in the same tissue or culture environment often relies on their differential gene expression. The presence or absence of such unique gene-products (‘markers’), allows for the determination of the cell’s identity (4). Changes in cellular identity can therefore be used to estimate the effect of environment alterations, cell culture time, and (lack of) exposure to certain growth factors on the efficacy and clinical safety of the tissue-engineered products. To obtain the most accurate prediction as possible, scientists are constantly searching to identify the most appropriate markers (1).

In the field of conjunctival tissue engineering, a standardized identification strategy or potential marker panel to characterize the cultured conjunctival epithelium has yet to be defined. Markers are required for the three main conjunctival cell populations; (I) stratified, squamous epithelial cells, (II) goblet cells, and (III) oligopotent stem cells (hereinafter referred to as progenitor cells) (5). Going onward, the current study will focus on markers for the first two populations (i.e., differentiated conjunctival cell types). To address the absence of a standardized marker panel, promising *in vivo* epithelial cell-specific markers should be investigated for their potential to identify their cultured counterpart. Supplementary Table 1 summarizes the mucins and keratins that are commonly expressed by the human conjunctiva.

The discriminative potential of mucins is associated with their functional categorization into three subgroups (i.e., secretory gel-forming mucins, secretory soluble mucins, and membrane-associated mucins (7)), which already hints at a cellular-specific expression pattern. The general rule of thumb is that the conjunctival stratified squamous epithelial cells express the membrane-associated mucins, while the goblet cells are considered the main source of secretory gel-forming mucins in mucosal epithelium (8, 9). This distinct cellular distribution applies for MUC1 and MUC5AC (Supplementary Table 2), turning these functional glycoproteins into promising candidates for our marker panel.

Keratins, on the other hand, are cytoplasmic intermediate filaments that are solely found in epithelial cells (10). Of the *in vivo* conjunctival keratins (Supplementary Table 1), both K13 and K19 are considered to be appropriate markers for squamous epithelial cells (Supplementary Table 2) (11). Some debate has, however, arisen on the previously accepted keratin marker K7 for conjunctival goblet cells (Supplementary Table 2). The conflicting characterization purpose of the OV-TL 12/30 clone of the K7 antibody perfectly illustrates the ongoing debate. This clone has been used to discriminate between corneal and conjunctival surface epithelium in general, while also been applied to specifically identify conjunctival goblet cells in cultured human conjunctival epithelium (12, 13). Hence, the cellular specificity for cultured conjunctival cells of K7 has yet to be defined.

In this study, groundwork will be provided for a robust identification panel to distinguish the cultured equivalents of the two differentiated conjunctival cell types through immunocytochemistry. The cellular specificity of the following markers will be investigated in cultured cells and in *in vivo* conjunctival cells; (I) K13, K19, and MUC1 for squamous epithelial cells and (II) K7, MUC5AC, and periodic-acid-Schiff (PAS)-positivity for goblet cells. The co-expression of K7/K19 will be evaluated as well to investigate the hypothesis implicating a resemblance between the conjunctiva (cfr., stratified, non-keratinized epithelium) and simple- and glandular epithelia (14–16). To conclude, this study will emphasize the power of immunocytochemistry to not only characterize tissue-engineered grafts but concomitantly asses its efficacy and clinical safety through the selection of appropriate markers.

## 2. Materials and methods

### 2.1. Tissue specimen

Human conjunctival tissue from 18 cadaveric donors was obtained from the Antwerp University Hospital tissue bank as ocular tissue rejects for clinical donation. The donor’s age ranged from 31 to 84 years, with an average of 66 years. Within 48 h post-mortem, the isolated conjunctiva was processed for frozen sections (*n* = 3), whole mount preparations (*n* = 3), *ex vivo* explant cultures [immunocytochemistry (*n* = 7); PAS staining (*n* = 4)] or both whole mount preparations and *ex vivo* explant cultures [immunocytochemistry (*n* = 1)] as described further on. To avoid non-conjunctival cell populations in the *ex vivo* explant cultures, conjunctiva was isolated from the bulbar region rather than the fornix or palpebral region. Furthermore, inferior and superior bulbar conjunctiva is routinely included to have a representation of divergent stem- and goblet cell densities in the samples (17, 18). The study followed the tenets of the Declaration of Helsinki and was approved by the Ethical Committee (EC) of the Antwerp University Hospital (approved EC: 11/2/12).

### 2.2. Primary human conjunctival epithelial cell cultures

Bulbar conjunctiva from the inferior and superior region was isolated and disinfected for 1 min in 0.5% povidone-iodine (Pharmacy of the Antwerp University Hospital, Edegem, Belgium), followed by a quadruple washing step in 1X phosphate buffered saline (PBS) [Life Technologies, Carlsbad, California, United States of America (USA)]. Primary human conjunctival cell cultures were initiated as previously illustrated (19). Briefly, the disinfected tissue was further cut into 2 mm ×2 mm explants and placed at the air-liquid surface to initiate outgrowth at 37°C and 5% CO_2_ for 14 days. The medium was changed thrice a week and consisted of keratinocyte serum-free medium (Life Technologies) supplemented with 50 μg/mL bovine pituitary extract (Life Technologies), 5 ng/mL recombinant human epidermal growth factor (Life Technologies), 10 μg/ml gentamicin (Life Technologies), and 1 μg/mL amphotericin B (Life Technologies). When a visible outgrowth was obtained, primary cultures were submerged. Explants were removed from culture upon the first signs of fibroblast outgrowth. After the culture period of 14 days, remaining explants were discarded and the primary cultures were fixed as described in ‘Histology and immunocytochemistry’.

### 2.3. Whole mount preparations and frozen sections

To obtain positive control samples, isolated conjunctival tissue was fixed in 4% paraformaldehyde (MLS nv, Menen, Belgium) for 30 min at 4°C, followed by a triple washing step with 1X PBS (Life Technologies). This fixed tissue corresponds to whole mount preparations. To additionally implement frozen sections as controls, fixed samples were also embedded in ‘Optimal Cutting Temperature’ medium (Sakura Finetek, Alphen aan den Rijn, The Netherlands) and stored at −80°C until cryosectioning. The corresponding cryostat sections were mounted on Superfrost microscope slides (VWR, Radnor, Pennsylvania, USA) and dried at 37°C for 2 h followed by their storage at −20°C and ultimately immunolabeling.

### 2.4. Histology and immunocytochemistry

The *ex vivo* conjunctival cultures were fixed using 4% paraformaldehyde (MLS) for 20min at 4°C, followed by a triple washing step with 1X PBS (Life Technologies). To visualize cultured goblet cells based on their stored (neutral) mucin content (20, 21), a PAS staining kit (Merck Millipore, Burlington, Massachusetts, USA) was used according to the manufacturer’s instructions. The two conjunctival cell types were further investigated by immunocytochemistry through the use of MUC1, MUC5AC, K7, K13, and K19 primary antibodies as described in Table 1. All primary and secondary antibodies were diluted in 1X PBS containing 10% normal goat serum, 0.01% bovine serum albumin (BSA), 0.05% thimerosal and 0.01% sodium azide (blocking buffer). Briefly, fixed cultures were permeabilized with 1% triton X-100 blocking buffer (30 min) and primary antibodies were incubated overnight at 4°C. Secondary antibodies were added for 2 h at 4°C (i.e., Cy3 /FITC-conjugated donkey-anti-rabbit antibody and Cy3/FITC-conjugated donkey-anti-mouse antibody (Jackson Immunoresearch, West Grove, Pennsylvania, USA), followed by a nuclear counterstain using 4′,6-diamidino-2-phenylindole (DAPI, Sigma-Aldrich, Saint Louis, Missouri, USA) for 1 min at room temperature. Samples were mounted in citifluor (Ted Pella 19,470, Redding, California, USA) and imaged on an Ultra*VIEW* VoX dual spinning disk confocal system (PerkinElmer, Waltham, Massachusetts, USA). The individual antibody specificity was evaluated using the MCF-7 cell line as positive control for all six antibodies. Negative staining controls for all immunocytochemical procedures were performed by substitution of non-immune sera for the primary or secondary antisera.

**Table 1:** Antibodies.

Antibodies per cell type	Specifications	Clone	Dilution	Company
Squamous epithelial cells					
Mucin 1	Rabbit polyclonal	n.a.	1/200	Abcam
Keratin 13	Rabbit monoclonal	EPR3671	1/500	Abcam
Keratin 19	Rabbit monoclonal	EP1580Y	1/200	Abcam
Goblet cells					
Mucin 5AC (ab3649)	Mouse monoclonal	45 M1	1/50	Abcam
Mucin 5AC (ab198294)	Rabbit monoclonal	EPR16904	1/500	Abcam
Keratin 7	Mouse monoclonal	OV-TL 12/30	1/50	Abcam

n.a., not applicable.

## 3. Results

**Figure 1:**
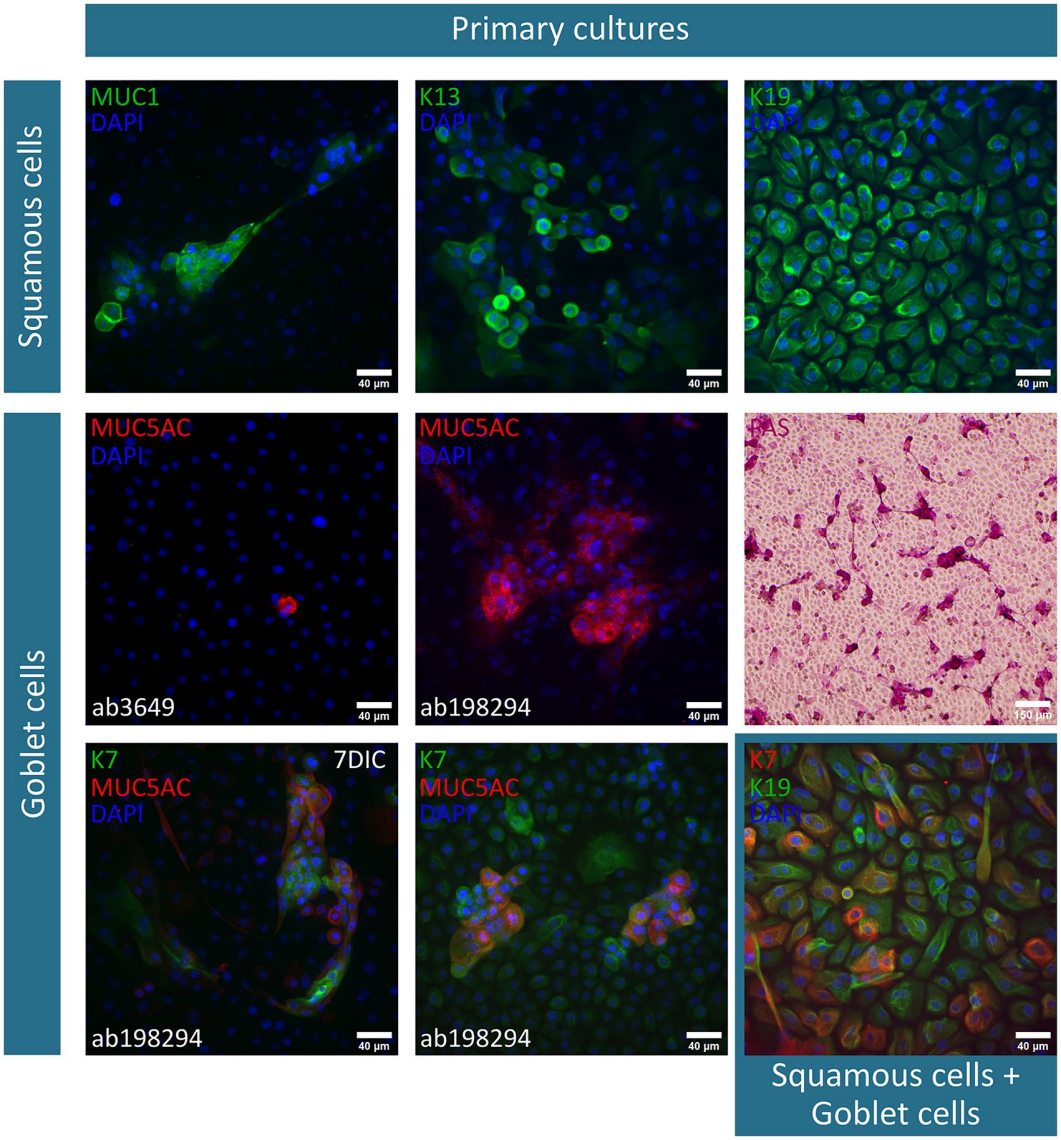
Immunofluorescent and periodic-acid-Schiff (PAS)-staining of primary conjunctival explant cultures after a two-week culture period. The investigated markers, along with their corresponding fluorescent label, are summarized per cell type (i.e., squamous epithelial cells and goblet cells) and depicted on each image. Nuclei are counterstained using 4′,6-diamidino-2-phenylindole (DAPI). Furthermore, deviations on the culture period [i.e., 1 week culture period instead of 14 days in culture (DIC)] or specification on the used mucin (MUC) 5AC antibody are mentioned. K, keratin.

**Figure 2:**
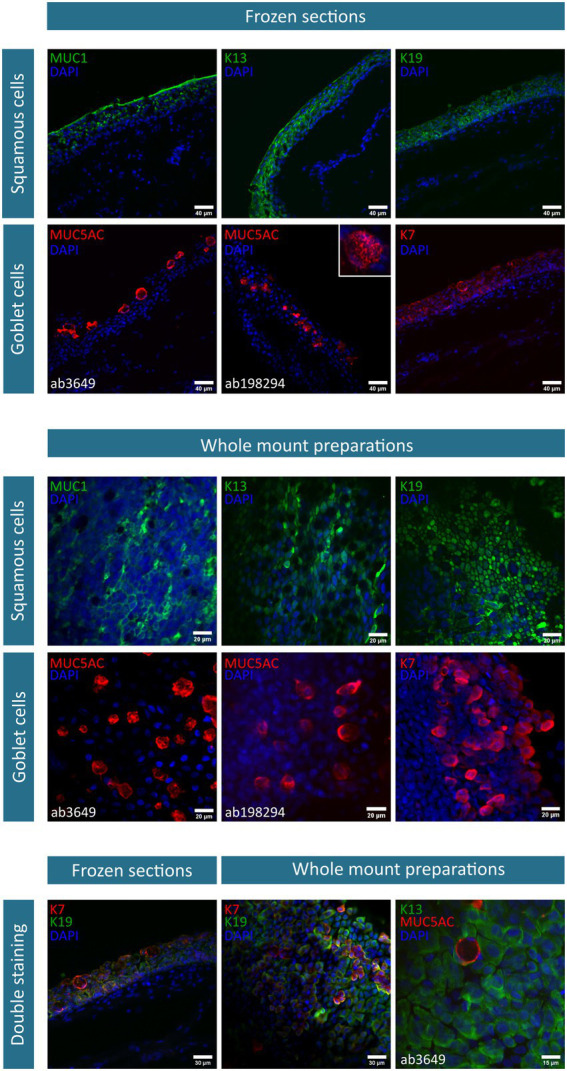
Immunofluorescent stainings of conjunctival frozen sections (*n* = 3) and whole mount preparations (*n* = 2). The investigated markers, along with their corresponding fluorescent label, are summarized per cell type (i.e., squamous epithelial cells and goblet cells) and depicted on each image. Nuclei are counterstained using 4′,6-diamidino-2-phenylindole (DAPI). As two mucin (MUC) 5AC antibodies are used, specifications are mentioned where necessary. Of note, the illustrative MUC5AC (ab198294) and K13/MUC5AC staining is exceptionally performed on one donor. K, keratin.

After 2 weeks of culture, the obtained cobblestone-like primary conjunctival cultures are characterized using different markers as shown in Figure 1. To verify our findings, the expression profile in control *in vivo* conjunctival samples (i.e., frozen sections of the conjunctiva (*n* = 3) and whole mount specimen (*n* = 2), Figure 2) was investigated and compared to our results and state-of-the-art literature. The observations related to each cell type will be discussed separately.

Squamous epithelial cells – This conjunctival cell type can be identified using MUC1 (*n* = 5), K13 (*n* = 4), and K19 (*n* = 6) in primary explant cultures and *in vivo* conjunctival samples. The expression of K19 is considered to be uniform in all cells of the conjunctival explant culture, while both MUC1 and K13 are expressed less abundantly in the suggested cultured squamous epithelial cells (Figure 1). In frozen sections of the conjunctiva, K19 is present in all cells throughout the different layers of the epithelium. MUC1 and K13 immunoreactivity is, instead, rather limited to the upper levels of the epithelium and devoid in round structures (Figure 2). Furthermore, when looking at the whole mount preparations, the presence of similar round, black ‘holes’ can be observed for the MUC1 and K13 stains (Figure 2). These unstained cells are reminiscent of conjunctival goblet cells.

Goblet cells – The overall mucin content of goblet cells was primarily visualized using a PAS-staining (*n* = 4). PAS^+^ cells in different shapes (i.e., round and elongated) and organizations (i.e., isolated and grouped) are observed throughout the culture (Figure 1). However, the clear presence of stored mucin by the PAS-staining did not align with the findings of MUC5AC-stained cells in the conjunctival explant cultures (ab3649, MUC5AC antibody, *n* = 5). Despite being the most abundant secreted conjunctival mucin, only occasionally an isolated MUC5AC^+^ cell was observed in a small fraction of explant cultures (Figure 1). The MUC5AC antibody (ab3649) was, however, able to identify MUC5AC produced by conjunctival goblet cells as it visualized large, round, inflated- or swollen-like cells in conjunctival frozen sections and whole mount specimens (Figure 2). With the use of a second MUC5AC antibody (ab198294), MUC5AC staining was better defined, showing small MUC5AC granules that were stored in conjunctival goblet cells of the frozen sections (Figure 2, insert frozen sections). In the conjunctival explant cultures, the ab198294 MUC5AC antibody was able to identify round cultured goblet cells that appeared as isolated or grouped cells as well as in some more elongated cells (Figure 1, *n* = 3). The observations of the ab198294 MUC5AC antibody were more in accordance with the PAS-staining.

Another known goblet cell marker that was used during this characterization study is the K7 marker (*n* = 4). K7 expression was found throughout the conjunctival epithelium in frozen sections, with a more pronounced intensity in the large, round apical cells that are reminiscent of goblet cells (Figure 2). On the contrary, a more restricted K7 expression was established in the whole mount specimen (Figure 2). To have a better understanding of K7 expression, a double staining with K19 was performed. Both in frozen sections and whole mount specimen, we found conjunctival cells whereby K7 as well as K19 expression dominated and cells having a similar K7/K19 expression (Figure 2). When K7 expression is determined in primary conjunctival cultures, a clear evolution could be observed (Figure 1). The relative amount of K7^+^ cells in the explant culture seemed to increase when going from 1 week in culture to 2 weeks in culture. Furthermore, the combination with MUC5AC revealed that K7 expression is undetectable or slightly present in some MUC5AC^+^ cells, while other cultured cells show a clear co-expression (Figure 1, 7 days in culture (DIC), *n* = 3). The co-expression of K7/K19 is additionally defined in conjunctival explant cultures (*n* = 4). Similar to the *in vivo* samples, K7^+^, K19^+^, and K7^+^/K19^+^ cells were observed (Figure 1).

## 4. Discussion

The characterization of cells and tissues often relies on several features; gene expression, protein production, functionality, and morphology (22). The ultimate goal is to define a selection of markers that is distinctive for one specific cell type (22). In this study, the accuracy of promising markers to distinguish squamous epithelial cells (i.e., MUC1, K13, and K19) and goblet cells (i.e., MUC5AC, K7, and PAS-staining) in human primary conjunctival explant cultures is evaluated based on marker localization, cellular morphology of positive cells, and expression pattern (Supplementary Table 2). The establishment of such a cell-specific immunostaining profile should allow to determine the differential identification and cellular organization of the explant cultures. Moreover, information on the functionality and clinical safety of the cultured conjunctival-derived cells could be linked to the morphological and phenotypic assessment.

Considering the evaluated squamous epithelial cell markers, both MUC1 and K13 are acknowledged as specific markers. In our hands, K19 emerged to be a more general conjunctival marker, based on (I) the K19 expression in distinctive round, large superficial cells in frozen conjunctival sections (i.e., presumed goblet cells) and (II) the absence of black ‘holes’ that are reminiscent of unstained goblet cells in whole mount preparations. The evaluation of goblet cell markers revealed, in turn, the inadequacy of K7 as specific marker as well as the importance of a correct subcellular location of the immunocytochemical-identified antigen (i.e., ab3649 MUC5AC antibody vs. ab198294 MUC5AC antibody). The classification of K7 as a non-specific conjunctival marker is supported by the observations of an overall K7 expression *in vivo*, the presence of MUC5AC^+^/K7^−^ cells in early culture periods (7 DIC), and the changing expression pattern throughout the culture period. Furthermore, we were unable to confirm a potential affiliation of the stratified, non-keratinized conjunctival epithelium with glandular or simple epithelia based on their shared K7 and K19 expression. As mentioned in the introduction, both glandular and simple epithelia are characterized with K7/K19 co-expression (14, 23). Both *in vivo* conjunctival cells and cultured cells, however, exhibit different expression patterns, i.e., predominantly K7 or K19 expression and K7/K19 co-expression. Hence, their expression patterns are hypothesized to be less related than initially thought.

The observed widespread conjunctival K7 expression in frozen sections are in line with previous reports challenging the use of K7 as a true goblet cell marker due to its broad expression in squamous epithelial cells (14, 24). It could be hypothesized that the difference in reactive pattern (i.e., exclusive vs. general staining) is the result of different antibody clones (cfr., OV-TL 12/30 clone was used in this study), tissue fixation, or processing. Methodological differences can induce conformation changes to the epitopes, epitope masking (i.e., limited epitope accessibility) or non-specific background (25). The study of Jirsova et al., however, already investigated this hypothesis through the implementation of three available clones of K7 antibodies (12). To correctly interpret their results, it is important to keep in mind that the stratified conjunctival epithelium is estimated to contain 0.58% progenitor cells and 5% goblet cells (17, 26). Despite attaining a reduced K7-positivity using other clones than the OV-TL 12/30 clone (i.e., approximately 25% of the superficial conjunctival cells in impression cytology samples) (12), this percentage still exceeds the estimated 5% of goblet cells. Hence, positivity in conjunctival squamous epithelial cells using other K7 antibody clones can additionally be confirmed.

Next, the observational accuracy of the changing expression pattern and its implications will be discussed in more depth. Overall, we observed a wide K7 distribution *in vivo* that evolves toward a reduced expression after a one-week culture period, followed by the return of a broad expression at 14 DIC. When keratins are used as characterization markers, it is important to note that these proteins are highly dynamic structures and that their pattern can be modified under certain circumstances (23). The rationale behind this dynamic nature is intertwined with their elaborated cellular function; going from mechanical support toward the modulation of several cellular processes such as growth, proliferation, differentiation, apoptosis, heat shock response, and organelle homeostasis (23, 27–29). When an explant is brought into culture, it resembles the following two situations where alterations in the keratin gene expression profile have already been described, i.e., wound healing and change in cellular substrate (30–33). It is therefore plausible that the ‘activated’ conjunctival cells at the edge of the explant also undergo changes in their keratin profile to acquire proliferative and migratory features as seen in wound healing (34). Moreover, the difference between the native conjunctival basement membrane and plastic culture ware surface can modify their keratin expression pattern even further. Based on our results, it seemed that culture initiation and progression solely impact the K7 expression. Of note, the presence of the specific K13 marker appears to be less affected during explant culture (data not shown).

As already mentioned above, the importance of an accurate and precise location has been strengthened through our observations regarding the use of two MUC5AC antibodies. In contrast to the ab3649 antibody, the ab198294 antibody was able to visualize MUC5AC-filled granules in frozen sections and was able to identify MUC5AC-filled goblet cells in conjunctival explant cultures. The initial discrepancy between the lack of MUC5AC^+^ goblet cells in culture using the ab3649 antibody and the abundance of PAS^+^ cells, encouraged us to further investigate the functionality of cultured goblet cells (35). An elaborated *in vitro* assessment to address true functional goblet cells has consequently emerged, including an evaluation of the different morphologies found in the conjunctival explant cultures (35). As mentioned in the introduction, it is preferable to select functional proteins as markers to obtain a preliminary indication of the grafts potency to reinstate ocular surface health. Our group, however, pinpointed the need to evaluate if an extrapolation of the protein’s presence toward the functionality of the corresponding cell can be carried out (35). When considering MUC5AC, it seems that the MUC5AC^+^ goblet cells at the end of the culture period are characterized with a deteriorating mucin production and secretion. Additional techniques are, therefore, required to confirm if the immunocytochemical-identified MUC5AC^+^ goblet cells are functional as well (35).

When establishing an immunocytochemical marker panel for *in vitro* cultures, one should be aware that the potential of a specific marker is influenced by several factors such as the culture protocol, staining procedure, and antibodies clones. The reliability of K7 to identify conjunctival goblet cells has, however, been questioned by several authors using different staining procedures and antibodies clones. Its limitation as goblet cell marker is gradually becoming a consensus. In case a promising *in vivo* marker is not expressed by the cultured equivalents, scientists are challenged to determine if the absence of expression is due to the culture protocol or staining procedure. Furthermore, in the event of the culture protocol results in a different expression pattern, it is crucial to elucidate the underlying cause and – if required – adapt the protocol accordingly.

To summarize, an *in vitro* cellular profile assessment of cultured conjunctival-derived cells in terms of morphology, phenotype, and cellular organization is proposed using a panel of markers for immunocytochemical stainings. Although a more thorough validation on a larger number of *ex vivo* explants might be required, our study already recommends the following markers for the characterization of *ex vivo* conjunctival cultures; (I) MUC1 and K13 for squamous epithelial cells and (II) MUC5AC and PAS-staining for goblet cells. K19 can be proposed as a general marker for conjunctival-derived cells.

## Data availability statement

The original contributions presented in the study are included in the article/Supplementary material, further inquiries can be directed to the corresponding author.

## Ethics statement

The study was approved by the Ethical Committee (EC) of the Antwerp University Hospital (approved EC: 11/2/12). Written informed consent was waived due to Article 12 of the Act of December 19, 2009 of the legislation of the Belgian law.

## Author contributions

SVA and IP: conceptualization and methodology. SVA: data acquisition, original draft preparation, and funding acquisition. SVA, BVdB, and IP: data interpretation. SVA, BVdB, MH, CK, and IP: review and editing. All authors have read and agreed to the published version of the manuscript.

## Funding

This research was funded by the Research Foundation Flanders (FWO), of which SVA obtained a personal PhD grant (FWO, grant numbers 1196418N and 1196420N).

## Conflict of interest

The authors declare that the research was conducted in the absence of any commercial or financial relationships that could be construed as a potential conflict of interest.

## Publisher’s note

All claims expressed in this article are solely those of the authors and do not necessarily represent those of their affiliated organizations, or those of the publisher, the editors and the reviewers. Any product that may be evaluated in this article, or claim that may be made by its manufacturer, is not guaranteed or endorsed by the publisher.
