# Supplementary material for: Immunocytochemical characterization of ex vivo cultured conjunctival explants; marker validation for the identification of squamous epithelial cells and goblet cells
Source: Front Med (Lausanne). 2023 Feb 27;10:1024926. doi: 10.3389/fmed.2023.1024926 (PMC10008928; doi:10.3389/fmed.2023.1024926)
Supplement: Supplementary file 1 [file Table_1.docx]

| Supplementary Table 1: Overview of general conjunctival discriminative characteristics | | | | | |
| --- | --- | --- | --- | --- | --- |
|  | **Mucins** | |  |  |  |
|  |  | Membrane-associated | MUC1, MUC4, MUC13, MUC15, MUC16, MUC17, MUC20 | | (1-5) |
|  |  | Secretory, gel-forming | MUC2, MUC5AC, MUC5B, MUC6, MUC19 | | (6-8) |
|  |  | Secretory, soluble | MUC7 | | (9-11) |
|  | **Keratins** | |  | |  |
|  |  | Major components | K4, K5, K7, K8, K13, K15, K19 | | (12, 13) |
|  |  | Minor components | K1, K6, K18 | | (12-14) |

Abbreviations used: K, keratin; MUC, mucin.

References

1. Inatomi T, Spurr-Michaud S, Tisdale AS, Gipson IK. Human Corneal and Conjunctival Epithelia Express Muc1 Mucin. *Invest Ophthalmol Vis Sci* (1995) 36(9):1818-27. Epub 1995/08/01.

2. Pflugfelder SC, Liu Z, Monroy D, Li DQ, Carvajal ME, Price-Schiavi SA, et al. Detection of Sialomucin Complex (Muc4) in Human Ocular Surface Epithelium and Tear Fluid. *Invest Ophthalmol Vis Sci* (2000) 41(6):1316-26. Epub 2000/05/08.

3. Woodward AM, Argueso P. Expression Analysis of the Transmembrane Mucin Muc20 in Human Corneal and Conjunctival Epithelia. *Invest Ophthalmol Vis Sci* (2014) 55(10):6132-8. Epub 2014/08/30. doi: 10.1167/iovs.14-15269.

4. Corrales RM, Galarreta D, Herreras JM, Saez V, Arranz I, Gonzalez MJ, et al. Conjunctival Mucin Mrna Expression in Contact Lens Wear. *Optom Vis Sci* (2009) 86(9):1051-8. Epub 2009/08/08. doi: 10.1097/OPX.0b013e3181b4f02e.

5. Argueso P, Spurr-Michaud S, Russo CL, Tisdale A, Gipson IK. Muc16 Mucin Is Expressed by the Human Ocular Surface Epithelia and Carries the H185 Carbohydrate Epitope. *Invest Ophthalmol Vis Sci* (2003) 44(6):2487-95. Epub 2003/05/27. doi: 10.1167/iovs.02-0862.

6. McKenzie RW, Jumblatt JE, Jumblatt MM. Quantification of Muc2 and Muc5ac Transcripts in Human Conjunctiva. *Invest Ophthalmol Vis Sci* (2000) 41(3):703-8. Epub 2000/03/11.

7. Yu DF, Chen Y, Han JM, Zhang H, Chen XP, Zou WJ, et al. Muc19 Expression in Human Ocular Surface and Lacrimal Gland and Its Alteration in Sjogren Syndrome Patients. *Exp Eye Res* (2008) 86(2):403-11. Epub 2008/01/11. doi: 10.1016/j.exer.2007.11.013.

8. Van Acker SI, Van den Bogerd B, Van Acker ZP, Vailionyte A, Haagdorens M, Koppen C, et al. Characterisation of Gel-Forming Mucins Produced in Vivo and in Ex Vivo Conjunctival Explant Cultures. *Int J Mol Sci* (2021) 22(19). Epub 2021/10/14. doi: 10.3390/ijms221910528.

9. Jumblatt MM, McKenzie RW, Steele PS, Emberts CG, Jumblatt JE. Muc7 Expression in the Human Lacrimal Gland and Conjunctiva. *Cornea* (2003) 22(1):41-5. Epub 2002/12/28. doi: 10.1097/00003226-200301000-00010.

10. Corrales RM, Calonge M, Herreras JM, Saez V, Chaves FJ. Human Epithelium from Conjunctival Impression Cytology Expresses Muc7 Mucin Gene. *Cornea* (2003) 22(7):665-71. Epub 2003/09/26. doi: 10.1097/00003226-200310000-00010.

11. Corrales RM, Narayanan S, Fernandez I, Mayo A, Galarreta DJ, Fuentes-Paez G, et al. Ocular Mucin Gene Expression Levels as Biomarkers for the Diagnosis of Dry Eye Syndrome. *Invest Ophthalmol Vis Sci* (2011) 52(11):8363-9. Epub 2011/09/21. doi: 10.1167/iovs.11-7655.

12. Merjava S, Neuwirth A, Tanzerova M, Jirsova K. The Spectrum of Cytokeratins Expressed in the Adult Human Cornea, Limbus and Perilimbal Conjunctiva. *Histol Histopathol* (2011) 26(3):323-31. Epub 2011/01/07.

13. Kasper M, Moll R, Stosiek P, Karsten U. Patterns of Cytokeratin and Vimentin Expression in the Human Eye. *Histochemistry* (1988) 89(4):369-77. Epub 1988/01/01.

14. Hughes JL, Lackie PM, Wilson SJ, Church MK, McGill JI. Reduced Structural Proteins in the Conjunctival Epithelium in Allergic Eye Disease. *Allergy* (2006) 61(11):1268-74. Epub 2006/09/28. doi: 10.1111/j.1398-9995.2006.01207.x.
