# Supplementary material for: Immunocytochemical characterization of ex vivo cultured conjunctival explants; marker validation for the identification of squamous epithelial cells and goblet cells
Source: Front Med (Lausanne). 2023 Feb 27;10:1024926. doi: 10.3389/fmed.2023.1024926 (PMC10008928; doi:10.3389/fmed.2023.1024926)
Supplement: Supplementary file 2 [file Table_2.docx]

| Supplementary Table 2: Selectivity and cellular localization of studied conjunctival markers | | | | |
| --- | --- | --- | --- | --- |
| Marker | | | Cell identification | Localization in conjunctiva |
|  | **Mucins** | |  |  |
|  |  | MUC1 | Squamous epithelial cells | mRNA distribution: throughout epithelium, absence in goblet cells (1) |
|  |  |  |  | Protein localization: apical, (supra)basal squamous cells depending on antibody (1) |
|  |  | MUC5AC | Goblet cells | mRNA distribution: goblet cells (2, 3) |
|  |  |  |  | Protein localization: cytoplasmic vesicles* (3) |
|  | **Keratins** | |  |  |
|  |  | K7 | Goblet cells, squamous epithelial cells (?) | Protein localization (impression cytology): goblet cells** (4) |
|  |  |  |  | Protein localization: squamous epithelial cells and goblet cells (5, 6) |
|  |  | K13 | Squamous epithelial cells | Protein localization: throughout epithelium, more present in superficial layers (5) |
|  |  | K19 | Squamous epithelial cells | Protein localization: throughout epithelium (5) |
| Abbreviations used: K, keratin; MUC, mucin.  *Secreted MUC5AC can be responsible for additional positivity due to its adherence to the epithelial glycocalyx (3).  ** K7 is used in single and double stainings to identify goblet cells in conjunctival biopsies, impression cytology samples, and cultured conjunctival epithelium (7-13). The implementation of K7 as single stain is practiced to identify goblet cells in general or to specifically quantify their total number (8, 10-12). The combination with MUC5AC or helix pomatia agglutinin in a double stain is performed to further specify between empty, round-shaped mature goblet cells, mucus-filled goblet cells, and even immature goblet cells (7, 9, 13). | | | | |

References

1. Inatomi T, Spurr-Michaud S, Tisdale AS, Gipson IK. Human Corneal and Conjunctival Epithelia Express Muc1 Mucin. *Invest Ophthalmol Vis Sci* (1995) 36(9):1818-27. Epub 1995/08/01.

2. Inatomi T, Spurr-Michaud S, Tisdale AS, Zhan Q, Feldman ST, Gipson IK. Expression of Secretory Mucin Genes by Human Conjunctival Epithelia. *Invest Ophthalmol Vis Sci* (1996) 37(8):1684-92. Epub 1996/07/01.

3. Jumblatt MM, McKenzie RW, Jumblatt JE. Muc5ac Mucin Is a Component of the Human Precorneal Tear Film. *Invest Ophthalmol Vis Sci* (1999) 40(1):43-9. Epub 1999/01/15.

4. Krenzer KL, Freddo TF. Cytokeratin Expression in Normal Human Bulbar Conjunctiva Obtained by Impression Cytology. *Invest Ophthalmol Vis Sci* (1997) 38(1):142-52. Epub 1997/01/01.

5. Merjava S, Neuwirth A, Tanzerova M, Jirsova K. The Spectrum of Cytokeratins Expressed in the Adult Human Cornea, Limbus and Perilimbal Conjunctiva. *Histol Histopathol* (2011) 26(3):323-31. Epub 2011/01/07.

6. Hughes JL, Lackie PM, Wilson SJ, Church MK, McGill JI. Reduced Structural Proteins in the Conjunctival Epithelium in Allergic Eye Disease. *Allergy* (2006) 61(11):1268-74. Epub 2006/09/28. doi: 10.1111/j.1398-9995.2006.01207.x.

7. Colorado LH, Pritchard N, Cronin BG, Efron N. Characterization of Goblet Cells in a Pterygium Biopsy Using Laser Scanning Confocal Microscopy and Immunohistochemistry. *Cornea* (2016) 35(8):1127-31. Epub 2016/07/09. doi: 10.1097/ICO.0000000000000902.

8. Tian L, Qu M, Wang Y, Duan H, Di G, Xie L, et al. Inductive Differentiation of Conjunctival Goblet Cells by Gamma-Secretase Inhibitor and Construction of Recombinant Conjunctival Epithelium. *Exp Eye Res* (2014) 123:37-42. Epub 2014/04/22. doi: 10.1016/j.exer.2014.04.001.

9. He H, Ding H, Liao A, Liu Q, Yang J, Zhong X. Effects of Mycophenolate Mofetil on Proliferation and Mucin-5ac Expression in Human Conjunctival Goblet Cells in Vitro. *Mol Vis* (2010) 16:1913-9. Epub 2010/10/30.

10. Moore JE, Vasey GT, Dartt DA, McGilligan VE, Atkinson SD, Grills C, et al. Effect of Tear Hyperosmolarity and Signs of Clinical Ocular Surface Pathology Upon Conjunctival Goblet Cell Function in the Human Ocular Surface. *Invest Ophthalmol Vis Sci* (2011) 52(9):6174-80. Epub 2011/04/27. doi: 10.1167/iovs.10-7022.

11. Shatos MA, Rios JD, Horikawa Y, Hodges RR, Chang EL, Bernardino CR, et al. Isolation and Characterization of Cultured Human Conjunctival Goblet Cells. *Invest Ophthalmol Vis Sci* (2003) 44(6):2477-86. Epub 2003/05/27.

12. Yokoo S, Yamagami S. Goblet Cell Differentiation Potential in Human Corneal Limbal Epithelial Progenitor Cells in Vitro. *Invest Ophthalmol Vis Sci* (2020) 61(12):27. Epub 2020/10/29. doi: 10.1167/iovs.61.12.27.

13. Chao C, Golebiowski B, Stapleton F, Zhou X, Chen S, Madigan MC. Conjunctival Muc5ac+ Goblet Cell Index: Relationship with Corneal Nerves and Dry Eye. *Graefes Arch Clin Exp Ophthalmol* (2018) 256(11):2249-57. Epub 2018/07/26. doi: 10.1007/s00417-018-4065-y.
